# The Structural and Spectral Features of Light-Harvesting Complex II Proteoliposomes Mimic Those of Native Thylakoid Membranes

**DOI:** 10.1021/acs.jpclett.2c01019

**Published:** 2022-06-16

**Authors:** Sam Wilson, Dan-Hong Li, Alexander V. Ruban

**Affiliations:** Department of Biochemistry, School of Biological and Behavioural Sciences, Queen Mary University of London, Mile End Road, London E1 4NS, United Kingdom

## Abstract

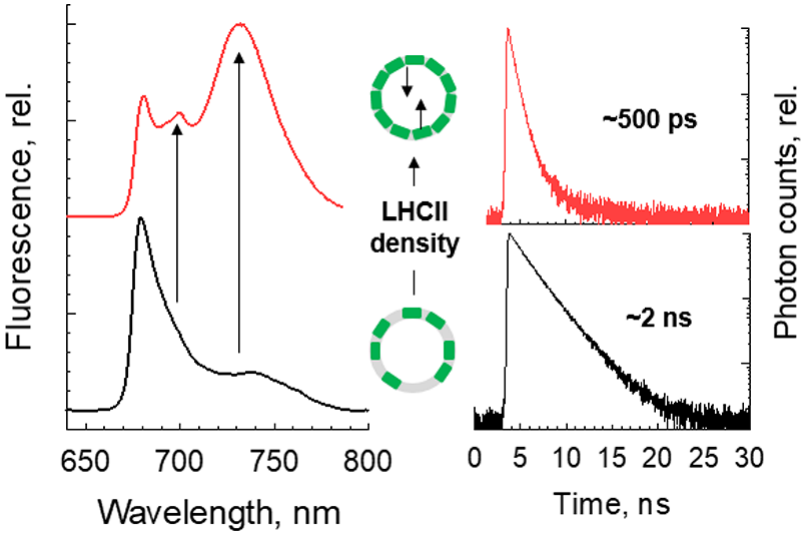

The major photosystem II light-harvesting antenna (LHCII) is the most abundant membrane protein in nature and plays an indispensable role in light harvesting and photoprotection in the plant thylakoid. Here, we show that “pseudothylakoid characteristics” can be observed in artificial LHCII membranes. In our proteoliposomal system, at high LHCII densities, the liposomes become stacked, mimicking the *in vivo* thylakoid grana membranes. Furthermore, an unexpected, unstructured emission peak at ∼730 nm appears, similar in appearance to photosystem I emission, but with a clear excimeric character that has never been previously reported. These states correlate with the increasing density of LHCII in the membrane and a decrease in its average fluorescence lifetime. The appearance of these low-energy states can also occur in natural plant membrane structures, which has unique consequences for the interpretation of the spectroscopic and physiological properties of the photosynthetic membrane.

Photosystems I (PSI) and II (PSII) harvest solar energy to generate oxygen and stable high-energy chemical compounds that are vital for life on Earth. Light-harvesting complex II (LHCII) plays a key role in light harvesting in green plants and algae, allowing efficient delivery of excitation energy into the reaction centers (RC) of the photosystems.^1^ Dynamic changes in the aggregation state of LHCII in the thylakoid membrane have been shown to be a significant fingerprint inherent to the regulation of light harvesting.^2−5^ Under illumination, photosynthetic electron transport induces a transthylakoid proton gradient, which acts in a negative feedback loop as the trigger to switch LHCII from a light-harvesting to photoprotective state, and thus of LHCII aggregation itself.^2,6^ These processes are further promoted by the low-pH activation of the PsbS protein and de-epoxidation of the LHCII-bound carotenoid violaxanthin into the more hydrophobic zeaxanthin.^2−4^ In this photoprotective state, excess absorbed light energy is dissipated as heat in a process termed energy-dependent nonphotochemical quenching (hereafter, qE).^3,7^ qE works to prevent photoinhibition of the PSII reaction center (RCII) and fine-tune the PSII quantum yield.

A fundamental technique for observing the energetic states of both photosystems has been to monitor chlorophyll (Chl) fluorescence emission at low temperatures where biochemical processes are arrested, the lifetimes of fluorescent species are longer, and emission peaks are more narrow and consequently more easily resolved.^8^ From these measurements, distinctive spectra can be observed. In plants, it is commonly accepted that the emission peaks at 680 nm (F680) and 700 nm (F700) arise from LHCII^9,10^ while peaks at ∼685 and ∼695 nm arise from the PSII core antenna proteins CP43 and CP47, respectively.^11^ A broad peak centered at ∼735 nm (F735) has been shown to be associated with PSI,^12^ with the red-most emission arising from the PSI light-harvesting antenna complex (LHCI).^13^ While these assignments are well accepted within the literature at large, there may be some overlap between the bands. Concomitant with the formation of the qE state and the quenching of the F680 band, an increase in the intensity of the F700 band has been observed at 77 K, and it is often used as a signature of LHCII aggregation.^14−16^ However, these states have also been readily observed under experimental conditions where aggregation is unachievable.^17−19^ Thus, it appears that these states are inherent to individual LHCII trimers, but the quenched conformational state, in which the F700 emission is prominent, is stabilized by aggregation.^19^ In this work, we have constructed LHCII-containing proteoliposomes with a range of protein densities. Static control of LHCII packing allows for the capture and stabilization of states that have been seen *in vivo* yet have eluded study due to the heterogeneous nature of complex natural systems.

Recent biochemical analysis has shown that there is an approximate 1:1 (gram:gram) lipid:Chl ratio in the PSII-enriched stacked grana of the thylakoid membrane.^20^ Accordingly, we decided to use this as one of the physiological extremes in the construction of the proteoliposomes, despite differences in bulk lipid composition. In our system, the average rate of incorporation of Chl into the membrane was 86.6 ± 6.5% of the initial starting amount, with no apparent dependence on the initial Chl:lipid ratio. Hereafter, any ratios in the text and figures represent final calculated Chl:lipid molar ratios. As the main comparison points, proteoliposomes with low (∼0.08:1) and high densities (∼0.80:1) of LHCII were primarily utilized.

Here, we show a typical spherical topology for the low-density proteoliposomes (Figure 1A). Each particle reached an average diameter of 59.2 ± 2.58 nm, with an average bilayer thickness of 3.63 ± 0.06 nm (Figure 1C,D). The bilayer thicknesses reported here are similar to those reported for *in vitro* and *in silico* DOPC membranes^21,22^ and *in vivo* thylakoid membranes.^23^ The high-density liposomes, however, display a largely divergent appressed morphology (Figure 1B). Particle sizes are larger than those of the low-density proteoliposomes at 75.8 ± 2.06 nm, with an apparent thickness of 6.05 ± 0.07 nm (Figure 1C,D). While the larger particle size may be ascribed to the greater LHCII:lipid ratio, the increase in the apparent thickness appears to indicate that the high-density proteoliposomes are stacked. Stacking of thylakoid grana membranes has been shown to be primarily reliant on the presence of LHCII^24−26^ and the net membrane surface charge.^26−30^ Interestingly, addition of Mg^2+^ to the high-density proteoliposomes appeared to promote stacking of the appressed membranes, further mimicking the *in vivo* grana structure (Figure S4). These data indicate that LHCII, a bilayer-forming lipid, and an appropriate net surface charge may be a minimal requirement for the formation of the classical thylakoid grana superstructure.

**Figure 1:**
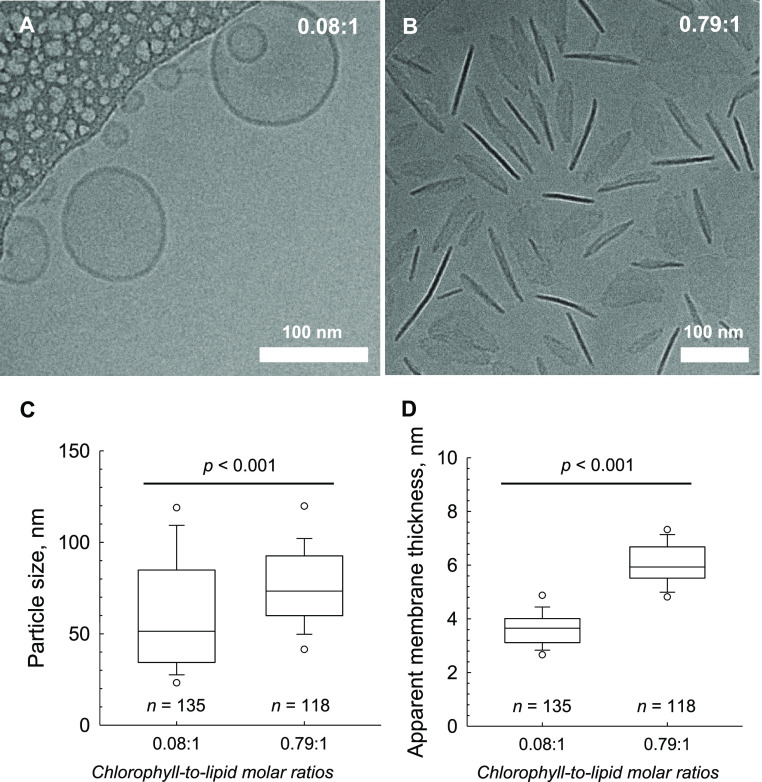
Microscopic analysis of LHCII proteoliposomes. (A) Representative cryo-EM micrograph of low-density LHCII proteoliposomes. The scale bar represents 100 nm. The 0.08:1 ratio represents the final chlorophyll:lipid molar ratio. (B) Representative cryo-EM micrograph of high-density LHCII proteoliposomes. The scale bar represents 100 nm. The 0.79:1 ratio represents the final chlorophyll:lipid molar ratio. (C) Particle sizes of low- and high-density proteoliposomes. Statistical significance determined via a Student’s *t* test. White dots represent 5th and 95th percentile values. The *x*-axis shows chlorophyll:lipid molar ratios. (D) Apparent membrane thicknesses of low- and high-density proteoliposomes. Statistical significance determined via a Student’s *t* test. White dots represent 5th and 95th percentile values. The *x*-axis shows chlorophyll:lipid molar ratios.

In detergent, LHCII has an average fluorescence lifetime (τ_avg_) of ∼4 ns.^31^ However, addition of LHCII to a liposome initially decreased the τ_avg_ to ∼2 ns, independent of protein–protein interactions in the membrane (Figure 2A), in line with previous reports.^32−39^ As the protein density in the liposome was increased, the τ_avg_ decreased progressively to ∼500 ps (Figure 2A and Supplementary Table 1). Here it is likely that stabilization of the energy-dissipative conformation of LHCII is promoted in environments in which the probability of LHCII aggregation is increased. Unexpectedly, however, the dominant feature of the 77 K emission spectra is the appearance of the broad, unstructured emission at ∼731 nm (hereafter, F730) that correlates with increasing LHCII density in the membrane (Figure 2C). Initially, the spectra of LHCII in a low-protein density membrane resemble the emission spectra seen in prior proteoliposomal studies,^40−42^ where the appearance of F700 appears to cause a broadening of the F680 peak. However, as the LHCII protein density is increased, the stoichiometry of F700 and F730 continues to increase similarly until a τ_avg_ of ∼1 ns is achieved (Figure 2B). After this point, F730 appears to become more dominant until the maximum τ_avg_ of ∼500 ps, where F700 is seemingly largely overlapped by it. At 77 K, the τ_avg_ of the F730 was ∼5 ns, similar to those in previous reports.^43^ Interestingly, at room temperature, there is only a slight difference in the bulk fluorescence spectra of the proteoliposomes (Figure S5). The absorption spectrum of low-density membranes reveals a blue-shifted chlorophyll *a* maximum indicative of either a change in chlorophyll environment or an alteration in pigment–protein or pigment–pigment interactions (Figure S5A). Such blue shifts are often accompanied by blue-shifted fluorescence. However, in this case, the fluorescence spectrum remained unchanged (Figure S5B). Therefore, it is possible that in the low-density membranes the pigment–pigment interactions were perturbed. This would result in a different excitonic profile, but without compromising either the efficiency of energy transfer to the terminal emitter or the state of the terminal emitter chlorophylls, such as their binding or environment. Furthermore, while the F700 band appears to poorly correlate with overall fluorescence quenching (*F*_q_) in this system, the relationship between *F*_q_ and the F730 band remains approximately linear (Figure 2D). Therefore, the amplitude of the F730 band appears to correlate strongly with LHCII quenching.

**Figure 2:**
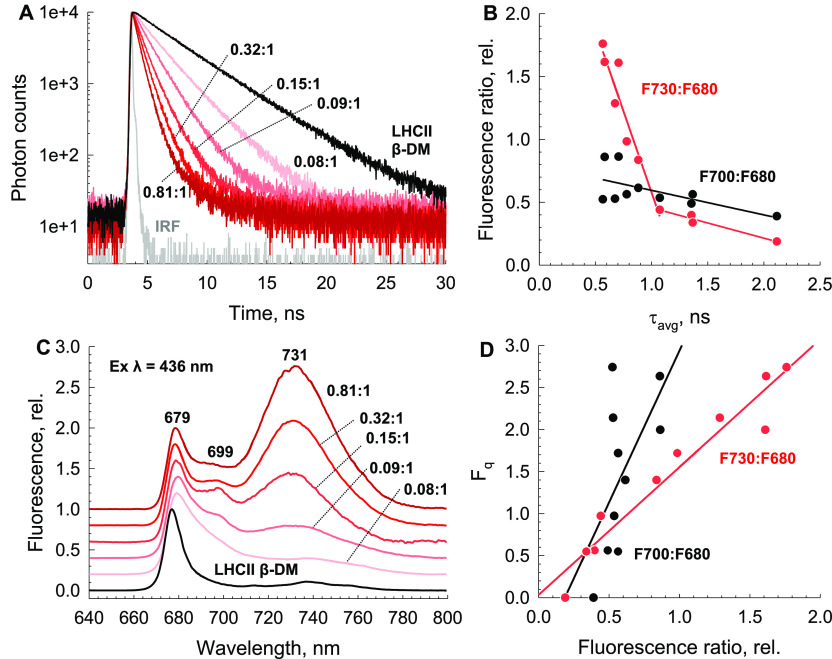
Appearance of the F730 band and its correlation with fluorescence lifetime and LHCII density in the proteoliposome. (A) 293 K fluorescence lifetimes of LHCII in detergent and a range of LHCII proteoliposomes with differing chlorophyll:lipid molar ratios. The excitation wavelength was 468 nm. Emission was measured at 680 nm. (B) Correlation between the average lifetimes (τ_avg_) of LHCII proteoliposomes and F700:F680 (black) and F730:F680 (red) 77 K fluorescence peak ratios. (C) 77 K fluorescence emission spectra of LHCII in detergent and a range of LHCII proteoliposomes with differing chlorophyll:lipid molar ratios. The excitation wavelength was 436 nm. Spectra are normalized to their respective F680 peak. (D) Correlation between 77 K fluorescence peak ratios and fluorescence quenching (*F*_q_): F700:F680 (black) and F730:F680 (red). Fluorescence quenching measured as (*F*_m_ – *F*_m_′)/*F*_m_′, where *F*_m_ represents the ∼2 ns average lifetime taken from the lowest-density proteoliposomes and *F*_m_′ is the measured average lifetime value of each proteoliposome preparation.

While the F700 band is commonly associated with LHCII, the far-red LHCII emission states have been seen in single-molecule spectroscopy (SMS) experiments, albeit at a much lower yield and frequency.^19,44^ Furthermore, these fluorescence states have been shown to be highly sensitive to external electric fields^45^ and show a strong temperature dependence,^43,46^ both of which are features of excited states with a strong charge transfer (CT) character.^47^ Similarly, large LHCII aggregates have been shown to display strong electron–phonon coupling in the red-most Chls, indicative of Chl–Chl CT character under such conditions.^48^ It has been hypothesized that the large red shifts in the fluorescence profile of LHCII are due to particular protein conformations that induce the aforementioned CT character that would otherwise have a low probability of access in detergent.^44^ Moreover, we have shown that the appearance of the F700 and F730 bands can be achieved through both detergent removal (Figure S6) and incubation in a glycerol-rich medium (Figure S7). Thus, it appears that environmental perturbations to the conformation of LHCII due to lipid–protein interactions, changes in the hydration state and salt content, and changes in specific protein–protein and packing dynamics in the membrane each can promote and stabilize the F700 and F730 emission sites, concurrent with quenching of τ_avg_.

As there are similar far-red emission bands in the LHCII proteoliposomes and PSI, we compared the fluorescence emission and excitation spectra of both with stacked thylakoid membranes (Figure 3). In each emission spectrum, the far-red region is dominated by a broad band (Figure 3A). In the excitation spectrum, for both PSI and thylakoid, the associated Chls display a broad absorption with maxima at ∼678 nm, with accompanying absorption in the far-red region up to ∼710 nm (Figure 3B). However, the LHCII proteoliposomes display a very different excitation spectrum. In this case, the maximum peak is blue-shifted by ∼4 nm to 674 nm, and the Chl *b* peak at 648 nm is much more prominent (Figure 3B). Most strikingly, the LHCII proteoliposomes display a sharp cutoff after the 674 nm maximum, with seemingly no corresponding far-red absorbing Chl, unlike the spectra for both PSI and the thylakoid membranes. This corresponds well to the absorption spectra, in which even the red-shifted Q_*y*_ absorption of the high-density proteoliposome is still blue-shifted with respect to that of isolated PSI (Figures S2 and S5). In PSI, the red-most absorption and emission bands arise from asparagine-mediated coordination of Chl *a*603 in the Lhca4 isoform of LHCI. Through site-directed mutagenesis, removal of this particular coordination results in a large blue shift of the absorption and emission to spectra that resemble those of LHCII.^49^ It is probable that subtle changes in the conformations of Chl dimers in both LHCI and LHCII are largely responsible for fine-tuning the absorption and emission characteristics, with a certain shared plasticity between each.^18^

**Figure 3:**
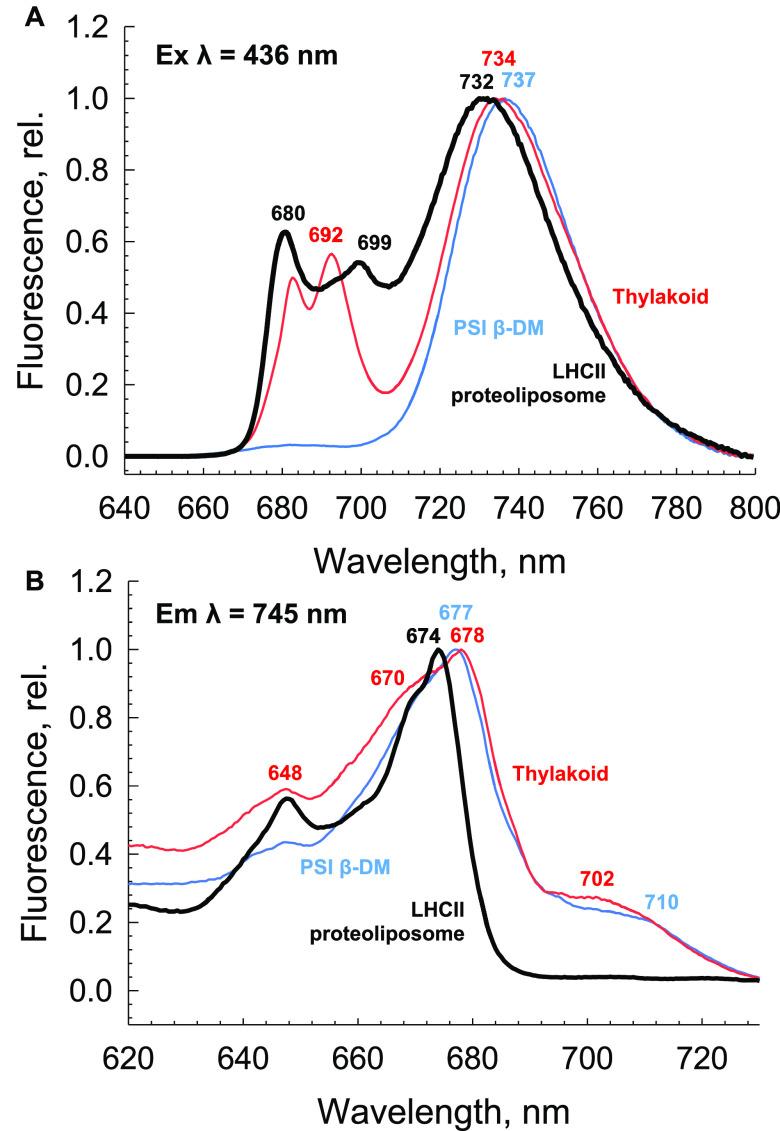
Identity of the F730 band through spectroscopic homology between isolated proteins and membranes. (A) 77 K fluorescence emission spectra of LHCII in a high-density proteoliposome (black), PSI in detergent (blue), and stacked spinach thylakoid membranes (red). The excitation wavelength was 436 nm. Spectra are normalized to their respective maxima. (B) 77 K excitation spectra for LHCII in a high-density proteoliposome (black), PSI in detergent (blue), and stacked spinach thylakoid membranes (red). The emission wavelength was 745 nm. Spectra are here normalized to their respective maxima.

However, in the LHCII proteoliposomes, the appearance of F730 emission (Figure 3A) does not correspond with any additional far-red absorption (Figure 3B), again in agreement with the observed bulk absorption spectra. This raises fundamental questions about the nature of these states. It is thus likely that the F730 state results from an excited state Chl–Chl or Chl–carotenoid interaction that does not exist in the ground state. In solution, similar behavior has been observed due to the formation of excimers or exciplexes, i.e., molecular dimers that are associated in the excited state yet dissociate during the transition to the ground state.^50,51^ These states result in an unstructured red-shifted emission that lacks a ground state absorption and were often proposed to originate from a mixture of CT and pure exciton states.^50,51^ Therefore, due to these features, the F730 band may likely arise from excimer or exciplex fluorescence in LHCII. While some reports have ascribed the F735 band in PSI to excimer fluorescence,^52^ the F735 band has associated far-red-absorbing chlorophylls that exist in the ground state, unlike the F730 band we have characterized in LHCII (Figure 3B). Hence, the F730 fluorescence appears to arise from chlorophylls that interact when excited yet exist as single monomers in the ground state. Even though the F730 and F735 bands appear with a similar fluorescence fingerprint, their ground state characteristics are highly divergent.

Mixed CT-exciton states are broadly accepted to be an intrinsic property of the PSI antenna protein LHCI,^52−54^ responsible for the majority of the broad F735 emission traditionally known as the fingerprint of PSI (Figure 3 and Figure S2). In LHCI, these states have been previously associated with a Chl *a/b* heterodimer located in the lutein (Lut) 2 site.^18,53,54^ Structural homology between the Lut2 sites in LHCI and LHCII had initially led to the hypothesis that the red-most emission states may arise from the Chl *a*603–*b*609–Lut2 locus in LHCII.^44^ While SMS measurements have shown that F700 does not correlate with fluorescence blinking,^19^ Stark fluorescence (SF) measurements on quenched LHCII show that F730 displays an inverse response to the electric field relative to F700, suggesting that the two states may act as competitive decay channels.^45^ Instead, the F730 state has been shown to strongly correlate with fluorescence blinking and increased qE conditions.^19^ These data have led to the suggestion that while F700 originates in the Lut2 locus, F730 arises from the terminal emitter Chl *a*610–*a*611–*a*612–Lut1 locus, which is also the putative site of qE.^19,55^ SF measurements have further identified far-red emission bands in the aggregated minor antenna proteins CP24 and CP26, each of which contains regions that resemble the Lut1 site of LHCII.^56^ Moreover, there is an absence of far-red emission bands in aggregated core antenna proteins where there are no loci analogous to the Lut1 domain of LHCII.^57^ Thus, the unique excimeric properties of F730, relative to F700 and F735, may arise from the environmental characteristics of the LHCII Lut1 locus.

The data presented thus far raise the questions of whether the LHCII F730 states exist *in vivo* and what physiological role these states may play. These far-red emission states may have been previously observed in *in vivo* systems but have mainly so far been ascribed to a population of PSI or uncoupled LHCI.^4,58−60^ To address this, we isolated thylakoid membranes and grana from wild type (WT) and lincomycin-grown (Linc-WT) *Arabidopsis thaliana* (*Arabidopsis*). Thylakoid membranes from plants grown on the chloroplast ribosome inhibitor lincomycin represent a physiological extreme where LHCII is largely overexpressed relative to RCII and PSI, similar to plants grown under low light.^24,32,61^ Relative to the WT thylakoids, we show that there is a large enrichment of LHCII in the isolated grana and Linc-WT membranes, relative to decreased levels of LHCI and PSI core complexes (Figure 4A and Figure S8). For the WT condition, this has an expected effect on the emission spectra, where the RCII-associated emission bands at 683 and 692 nm are largely unaffected, while there is a large decrease in the far-red emission and a subtle reduction in the F680 shoulder (Figure 4B and Figure S9). However, in the Linc-WT grana, the emission spectrum is dominated by the appearance of F730 emission, despite the reduction in overall PSI content. Furthermore, the blue shift of F730 in the Linc-WT grana relative to the WT grana may reflect a smaller contribution of F735-emitting species. Moreover, the presence of RCII in the WT grana may act to prevent formation of larger LHCII aggregates, diminishing the predominance of the F730-emitting states.

**Figure 4:**
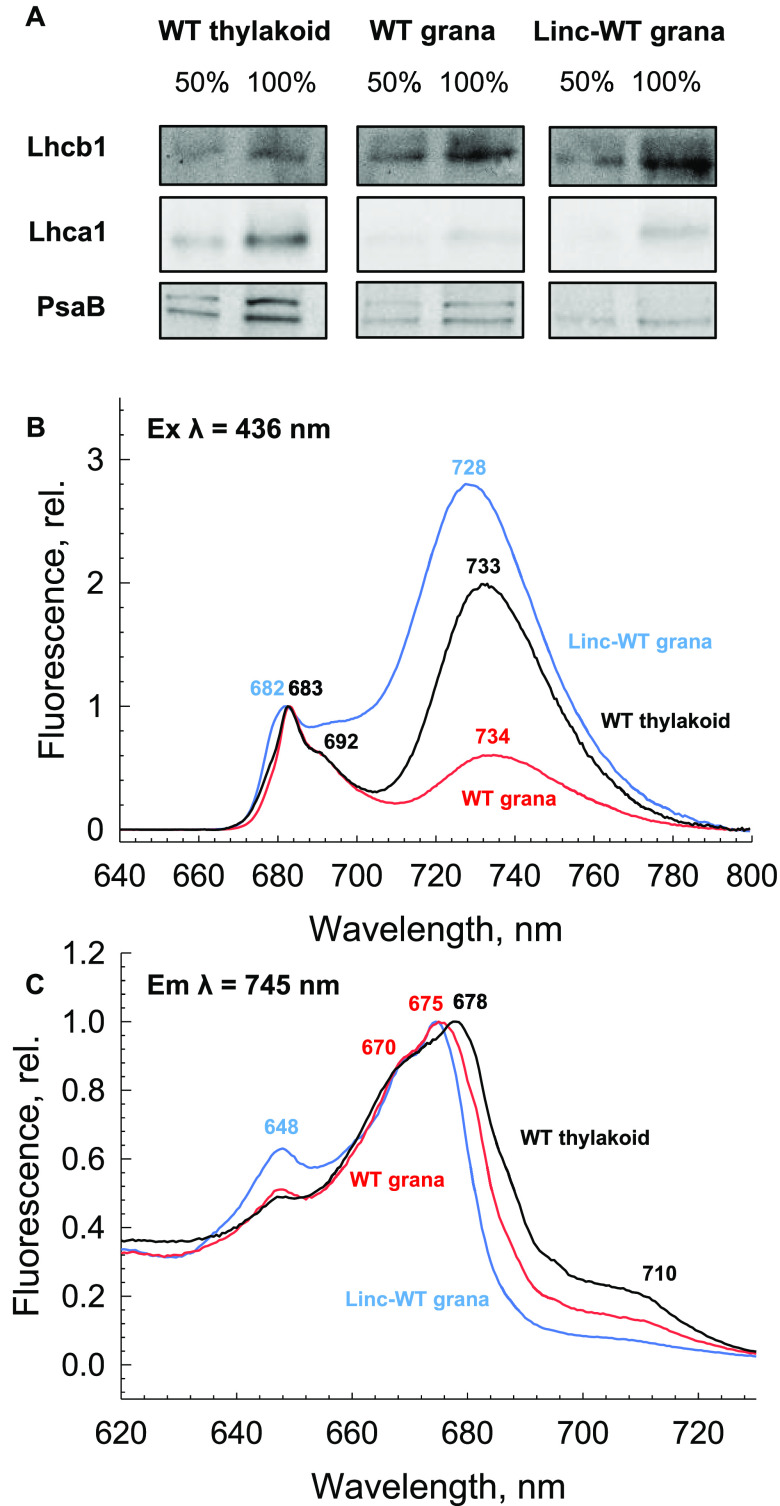
F730 band *in vivo*. (A) Western blot analysis of Lhcb1, Lhca1, and PsaB in WT *Arabidopsis* thylakoid membranes, WT *Arabidopsis* grana membranes, and Linc-WT *Arabidopsis* grana membranes. 100% corresponds to 3 μg of total chlorophyll mL^–1^. (B) 77 K fluorescence emission spectra of WT *Arabidopsis* thylakoid membranes (black), WT *Arabidopsis* grana membranes (red), and Linc-WT *Arabidopsis* grana membranes (blue). The excitation wavelength at 436 nm. Spectra are normalized to their respective maxima at ∼680–685 nm. (C) 77 K excitation spectra of WT *Arabidopsis* thylakoid membranes (black), WT *Arabidopsis* grana membranes (red), and Linc-WT *Arabidopsis* grana membranes (blue). The emission wavelength was 745 nm. Spectra are normalized to their respective maxima.

To try to deconvolute the nature of these bands, we further measured excitation spectra for the far-red emission under each condition (Figure 4C and Figure S9). The WT thylakoid spectrum again reflects a predominant contribution of PSI to the far-red emission, with a maximum located at 678 nm and a broad tail into the far-red region (Figures 3B and 4C). Similarly, the WT grana excitation still displays a far-red absorption tail, albeit at a lower amplitude. However, here the maximum is blue-shifted to 675 nm, with a slight increase in the Chl *b*-associated 648 nm band, each feature likely indicative of a stronger influence of LHCII on the emission spectrum. Interestingly, there are noteworthy differences in the excitation spectrum for the Linc-WT F730 band. Here, the far-red absorption tail is nearly absent, the maximum is blue-shifted to 674 nm, and the 648 nm peak becomes much more dominant. This again likely indicates an overwhelming contribution of LHCII to the large F730 seen in these membranes. While these emission states do likely represent an admixture of LHCI/PSI and LHCII, it is clear that the amplitude of F730 may not correlate with PSI content and may be influenced by the LHCII packing conditions, even in *in vivo* membranes. Thus, particular care should be taken in the physiological analysis of 77 K emission spectra, for example in the analysis of state transitions or the PSII:PSI ratio. This may be an issue in transgenic organisms or under growth conditions where LHCII is principally affected.

As the appearance of F730 is strongly linked to quenching of LHCII, its relevance to qE remains a key question. In previous studies on WT^15^ and lincomycin-grown plants^43,60^ and native LHCII aggregates,^4^ the F730 band appears to become largely quenched in the qE state. Thus, it is unlikely that this state plays a role as a direct quencher itself. As the putative location of the F730 band is shared with the qE locus in the LHCII Lut1 site,^19^ alterations in the coupling of the Lut1 S_1_ state to the Chl *a*611–*a*612 homodimer in the qE state may quench F730 emission. Therefore, we propose that F730 may be an indicator of an LHCII aggregation state and environment tuned for qE.

To summarize, we have constructed a range of minimal LHCII proteoliposomes that display variable characteristics that resemble the thylakoid as a whole. High densities of LHCII in the membrane cause stacking of the proteoliposomes, which can form larger superstructures that resemble the *in vivo* grana membranes of plants. Furthermore, we have shown that the specific conformational substates of LHCII that display the F700 and F730 states are promoted by aggregation in the proteoliposomal membrane. These states are likely due to specific environmental alterations to the structure of LHCII and the pigment–pigment interactions therein. Despite initial similarities to F735 of PSI, we have shown F730 to be distinct in nature and present in natural membranes under certain conditions. We suggest that F730 results from excimer fluorescence originating from the pigments of the LHCII Lut1 site. Thus, this is the first direct evidence of the occurrence of such associates within LHCII complexes that can be established only in the excited state, a feature of a true localized exciton.

## Experimental Methods

*Plant Material and Growth Conditions*. Spinach (*Spinacia oleracea*) was obtained from a local market and adapted to the dark for 45 min prior to any experiment. WT *Arabidopsis* (*Arabidopsis thaliana*; Col-0) seeds were sterilized in 50% (v/v) ethanol and 0.1% (v/v) Triton X-100 and stored for 48 h at 4 °C before being sown on a 6:6:1 Levington M3 compost/John Innes No. 3 soil/Perlite mixture (Scotts). Control experiments on WT *Arabidopsis* were carried out on 4–5-week-old plants grown at 200 μmol of photons m^–2^ s^–1^, with a 10 h photoperiod at 22 °C. Lincomycin-treated WT plants (Linc-WT) were treated as previously described,^32^ under the same growth conditions as control plants. Plants were grown in a Percival AR-75L3 plant growth cabinet (Percival Scientific Inc.) equipped with Phillips MASTER TL-D Super 80 36 W/840 bulbs, which emit a cool white light (Koninklijke Philips N.V.). Before each experiment, plants were adapted to the dark for 45 min.

*Sample Preparation and Biochemical Analysis*. Stacked thylakoid membranes were isolated as previously described,^4^ as were isolated grana membranes.^20^ LHCII was isolated from PSII-enriched spinach BBY particles,^62^ using a ratio of 0.6% (w/v) β-DM to 1 mg of Chl mL^–1^ to solubilize; PSI was similarly isolated from spinach stacked thylakoid membranes, using a ratio of 1% (w/v) β-DM to 1 mg of Chl mL^–1^ to solubilize. Either solubilized protein extract, at a concentration of 250–300 μg of total Chl, was loaded on a seven-step 0.15 to 1 M exponential sucrose density gradient and centrifuged for 18–20 h at 40 000 rpm and 4 °C, as previously described.^63^ The protein purity was confirmed through the characteristic absorption and fluorescence spectra (Figures S1 and S2) and sodium dodecyl sulfate–polyacrylamide gel electrophoresis (SDS–PAGE) analysis (Figure S3). Western blot and SDS–PAGE experiments were undertaken on isolated proteins and membranes as previously described,^4,64^ with equal amounts of total Chl (1–3 μg) loaded onto each lane per sample. Chl was quantified as described previously.^65^ Prior to any experiment, the isolated protein complex was run through a PD-10 desalting column into a buffer containing 10 mM NaCl, 20 mM HEPES (pH 7.6), and 0.03% (w/v) β-DM [or 0.01% (w/v) β-DM for overnight glycerol incubation, as in Figure S7]. The pigment composition was further analyzed through reverse-phase HPLC (Supplementary Table 2) using a LiChrospher 100 RP-18 column with a 5 μm particle size (Merck) and a Dionex Summit chromatography system (Dionex), as previously described.^66^

*In vitro* aggregation of LHCII was achieved using BioBeads SM-2 resin (Bio-Rad), as described previously.^55^ For glycerol incubation experiments, LHCII was incubated in a buffer containing 70% (v/v) glycerol, 10 mM NaCl, 20 mM HEPES (pH 7.6), and 0.01% β-DM for 20 h at 4 °C. For all other experiments, no sample was left in a glycerol medium for more than 1 h to avoid artifacts induced by a glycerol-rich solvent.

Proteoliposomes were formed in a manner similar to multiple previously published protocols,^40,41,67−70^ with the following minor alterations. Dipalmitoylphosphatidylcholine (DOPC) in chloroform (Avanti Polar Lipids) was placed in a glass vial and with the solvent evaporated under a steady flow of N_2_, while being rotated to form a thin film. The film was then resuspended in a buffer containing 20 mM HEPES (pH 7.6) and 10 mM NaCl for a final DOPC concentration of 1 mg mL^–1^. The resulting solution was vortexed for 2 min and subsequently passed through a 100 nm pore extruder, as per the manufacturer’s instructions (Avanti Polar Lipids). Then, 0.03% (w/v) β-DM was added to the lipid solution and the mixture incubated at 4 °C for 15 min. Isolated LHCII was then added at the required Chl:lipid ratio and sonicated in a water bath for 5 min. To remove detergent from the LHCII/lipid liposomes, 45 mg mL^–1^ BioBeads SM-2 resin (Bio-Rad) was added and the mixture incubated while rotating for 2 h at room temperature, followed by an additional 45 mg mL^–1^ overnight at 4 °C. BioBeads were then removed by running the resultant mixture through one layer of muslin cloth, before centrifugation at 15 000 rpm for 15 min at 4 °C to pellet any LHCII aggregates that were not incorporated into proteoliposomes. Thereafter, the proteoliposomal solution was separated from the large aggregates and stored at 4 °C. Chl was quantified in the pellet and proteoliposomal solution, as previously described.^65^

*Cryo-Electron Microscopy*. For visualization, proteoliposomes were loaded onto a lacey grid and plunge frozen using a Leica EM GP2 instrument (Leica Microsystems) before imaging on a JEOL JEM-2100plus electron microscope (JEOL) equipped with a OneView Gatan camera (AMETEK) using the SerialEM software package.^71^ Images were analyzed using ImageJ.^72^ Statistical significance between results was determined using a Student’s *t* test. For experiments that examined the stacking of the proteoliposomes, 5 mM MgCl_2_ was added to the final preparation of high-density proteoliposomes and the mixture incubated for 1 h prior to grid preparation.

*Spectroscopic Analysis*. Steady state fluorescence spectra were recorded for samples at a concentration of 6 μg of total Chl mL^–1^ using a FluoroMax-3 spectrofluorimeter (HORIBA Jobin Yvon, Longjumeau, France). The 77 K measurements were taken in 1 cm path length cuvettes in an Optistat DN liquid nitrogen-cooled bath cryostat (Oxford Instruments). Protein and proteoliposome samples were resuspended in a buffer containing 70% (v/v) glycerol, 10 mM NaCl, and 20 mM HEPES (pH 7.6), with 0.03% (w/v) β-DM added to the buffer for isolated protein, while *in vivo* membrane samples were resuspended in a reaction medium containing 70% (v/v) glycerol, 5 mM MgCl_2_, 0.33 M sorbitol, and 20 mM HEPES (pH 7.6). For emission spectra, samples were excited at 436 nm and emission was detected between 600 and 800 nm, with the detector defined by a long-pass filter with a sharp cutoff at 650 nm. Excitation spectra of the F730 band were recorded at 745 nm and measured from 620 to 735 nm, with the detector defined by a long-pass filter with a sharp cutoff at 715 nm. The optical path length was 1 cm. The spectral correction was applied within the FluorEssence software, according to the manufacturer’s specifications (HORIBA Jobin Yvon).

Time-correlated single-photon counting (FluoTime 200 Fluorimeter, PicoQuant) measurements were performed to quantify the fluorescence lifetime of a sample. Samples were excited at 468 nm using a 0.6 mW laser diode at a 20 MHz repetition rate, with fluorescence detected at 680 nm with a 2 nm slit width. The optical path length was 1 cm. All samples were measured at a concentration of 6 μg of total Chl mL^–1^. This setup has been previously demonstrated to have a negligible probability of singlet–singlet annihilation artifacts.^35^ These data were analyzed using the FluoFit software (PicoQuant), with the χ^2^ parameter and autocorrelation functions used to assess the quality of the fit. Average lifetimes were calculated as described previously.^64,73^ Fluorescence quenching was calculated as *F*_q_ = (*F*_m_ – *F*_m_′)/*F*_m_′, where *F*_m_ and *F*_m_′ are the maximum fluorescence yields in the dark and light, respectively. *F*_m_ was taken as the ∼2 ns lifetime taken from the 0.08:1 proteoliposomes, corresponding to the *F*_m_ fluorescence lifetime previously reported for LHCII in intact thylakoid membranes.^32−39^

Room-temperature absorption spectra were recorded on samples at a concentration of 6 μg of total Chl mL^–1^ on a modernized Aminco DW-2000 UV–vis spectrophotometer (Olis Inc.), with an *x*-axis resolution of 1 nm. The optical path length was 1 cm.

## Supporting Information Available

Spectroscopic and biochemical analysis of isolated LHCII and PSI, extended microscopic analysis of proteoliposomes, room-temperature fluorescence and absorption spectra, observation of the F730 band in aggregated LHCII and glycerol-incubated LHCII, biochemical and spectroscopic analysis of WT and Linc-WT thylakoid and grana, lifetime component analysis of proteoliposomes, and HPLC analysis of isolated LHCII (PDF)
