# Supplementary material for: The Structural and Spectral Features of Light-Harvesting Complex II Proteoliposomes Mimic Those of Native Thylakoid Membranes
Source: J Phys Chem Lett. 2022 Jun 16;13(24):5683–91. doi: 10.1021/acs.jpclett.2c01019 (PMC9237827; doi:10.1021/acs.jpclett.2c01019)
Supplement: Supplementary file 1 — jz2c01019_si_001.pdf [file jz2c01019_si_001.pdf]

Supporting Information

Structural and Spectral Features of Light-Harvesting Complex II Proteoliposomes Mimic Native Thylakoid Membranes

Sam Wilson, Dan-Hong Li, and Alexander V. Ruban*

Department of Biochemistry, School of Biological and Behavioural Sciences, Queen Mary University of London, Mile End Road, London, E1 4NS, United Kingdom

*corresponding author: a.ruban@qmul.ac.uk

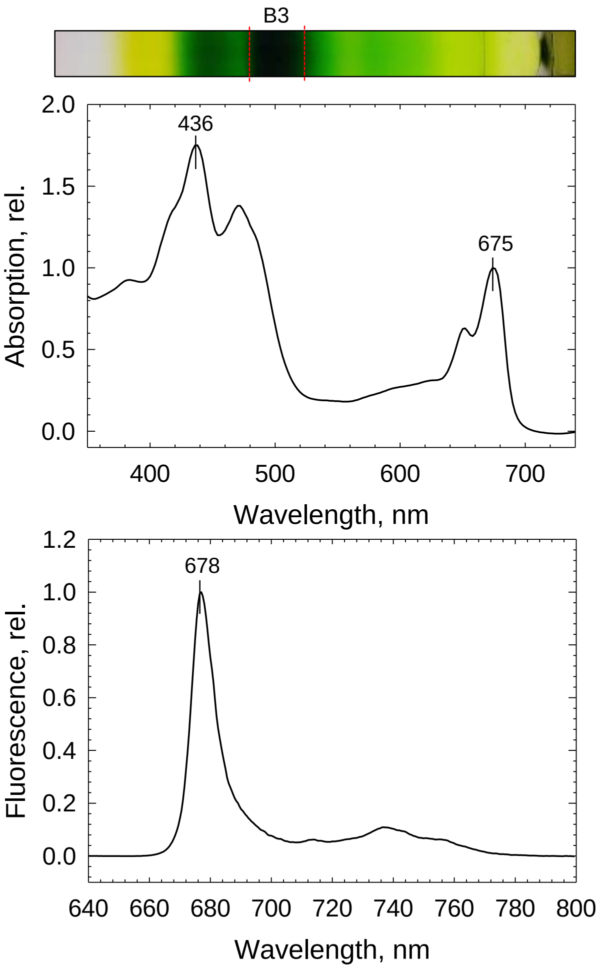

Fig. S1 ***Isolation of LHCII from PSII-enriched BBY particles***

(A) Sucrose gradient profile of solubilised BBY particles. B3 is highlighted for further analysis.

(B) 293 K absorption spectra of isolated LHCII in detergent. Data has been normalised to its Q_y maximum.

(C) 77 K fluorescence emission spectra of isolated LHCII in detergent. Excitation wavelength was at 436 nm. Data has been normalised to its maximum.

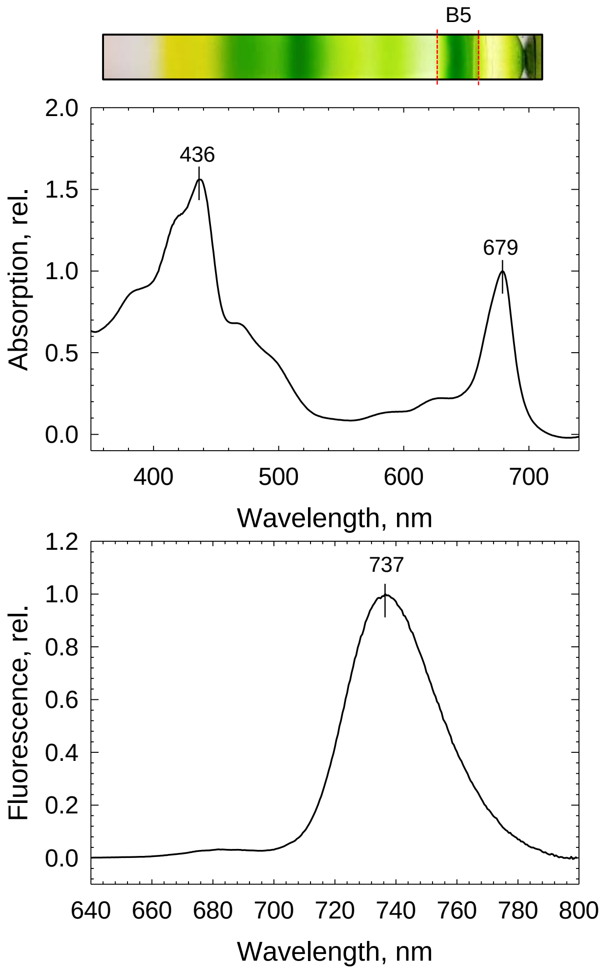

Fig. S2 ***Isolation of PSI from stacked thylakoid membranes***

(A) Sucrose gradient profile of thylakoid membranes. B5 is highlighted for further analysis.
(B) 293 K absorption spectra of isolated PSI in detergent. Data has been normalised to its Q_y maximum.
(C) 77 K fluorescence emission spectra of isolated PSI in detergent. Excitation wavelength was at 436 nm. Data has been normalised to its maximum.

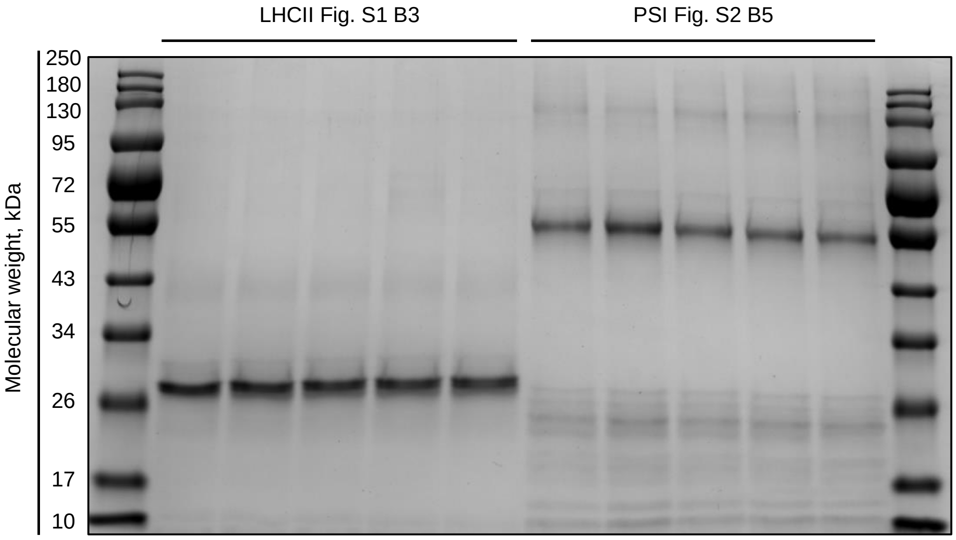

Fig. S3 ***SDS-PAGE of isolated LHCII and PSI bands***
12% Tris-glycine SDS-PAGE gel stained with InstantBlue Coomassie protein stain. Left-hand side lanes show replicates of the B3 band highlighted in Fig. S1; right-hand side lanes show replicates of B5 band highlighted in Fig. S2. Loaded total chlorophyll was between 1 – 3 µg.

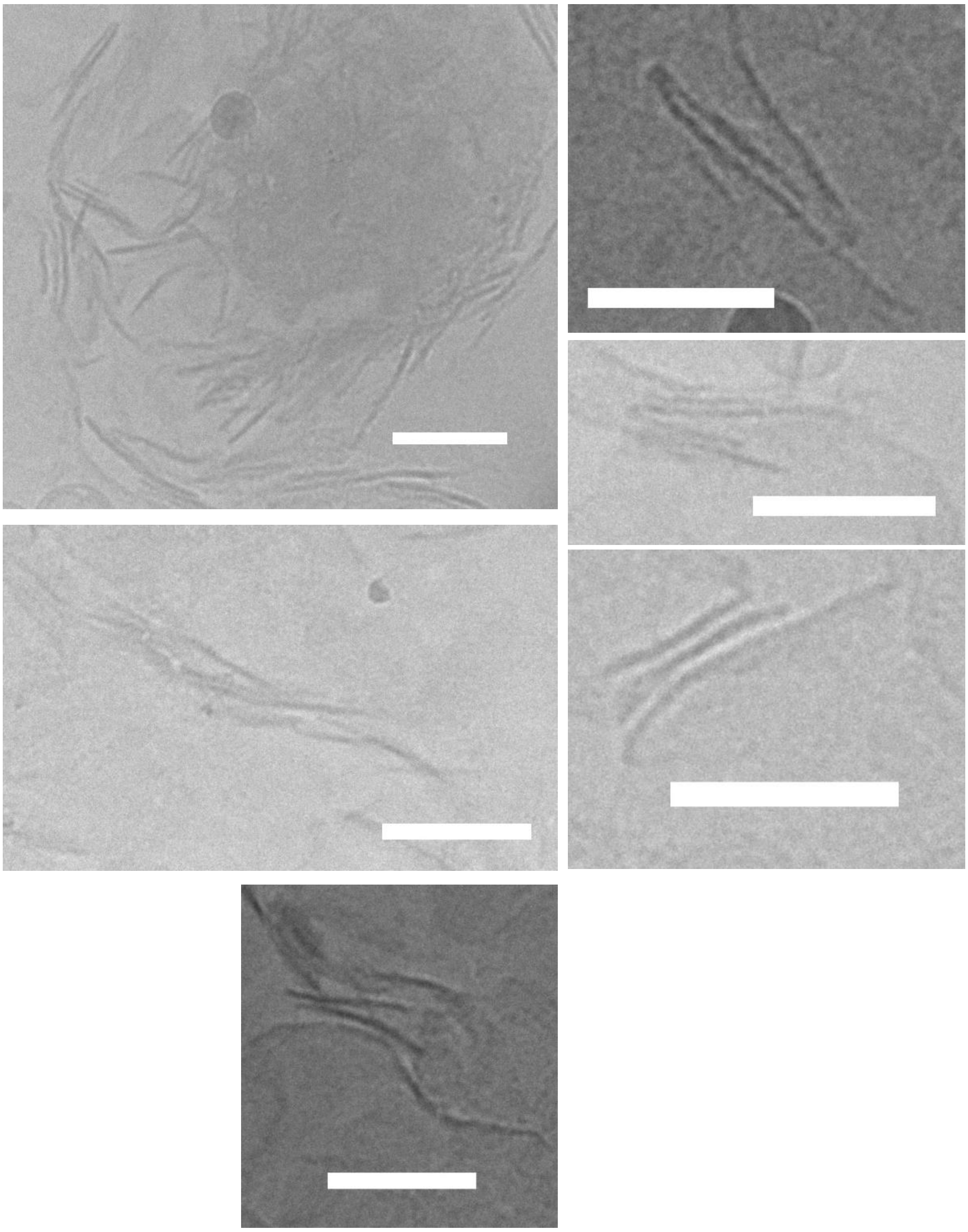

Fig. S4 ***Microscopic analysis of addition of Mg^{2+} to the high-density proteoliposomes***
Representative micrographs from cryo-EM experiments where $MgCl_2$ was added to the high-density proteoliposomes and incubated for 1 h at 4°C. Scale bar represents 100 nm.

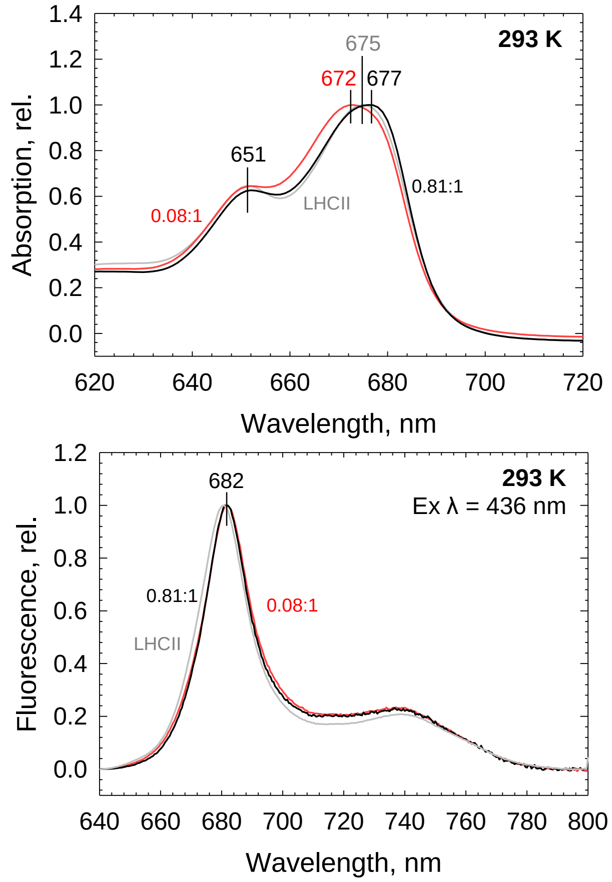

Fig. S5 ***Comparison of both low- and high-density LHCII proteoliposomes with isolated LHCII at 293 K***

(A) 293 K absorption spectra of isolated LHCII in detergent (grey), high-density LHCII proteoliposome (black), and low-density proteoliposome (red). Ratios are measured chlorophyll-to-lipid ratios. Spectra are normalised to their Q_y maxima.

(B) 283 K fluorescence emission spectra of isolated LHCII in detergent (grey), high-density LHCII proteoliposome (black), and low-density proteoliposome (red). Ratios are measured chlorophyll-to-lipid ratios. Excitation wavelength was at 436 nm. Spectra are normalised to their maxima.

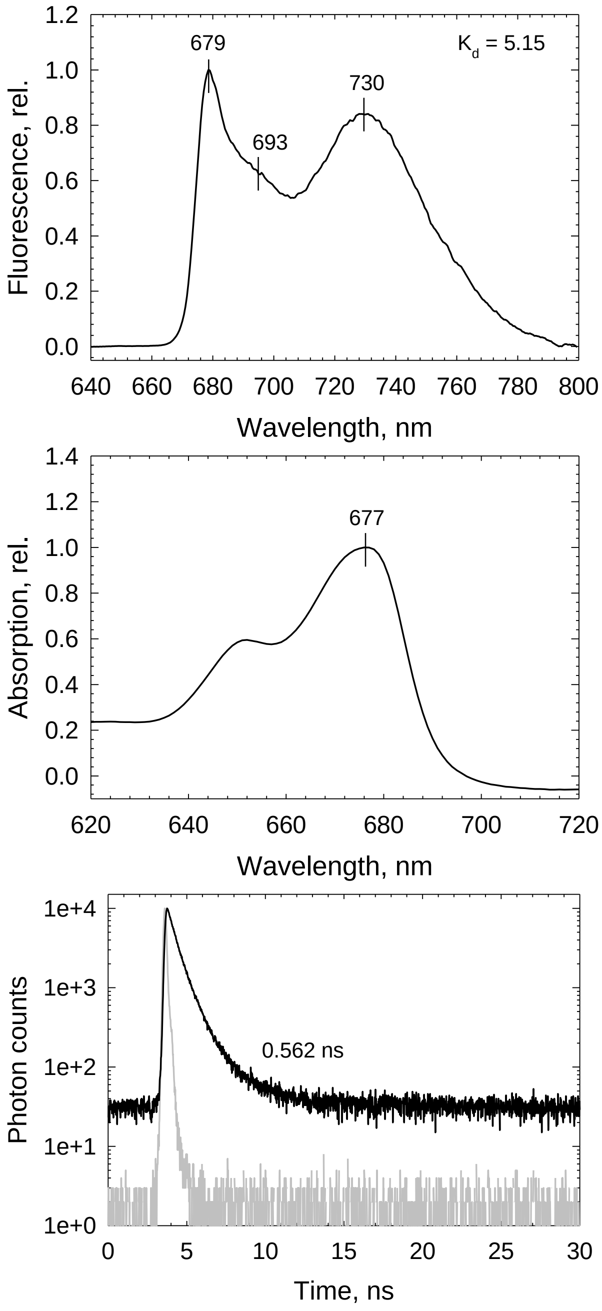

Fig. S6 ***Observation of the F730 band in LHCII aggregated in solution via detergent removal***

(A) 77 K fluorescence emission spectra of LHCII aggregates. Excitation wavelength was at 436 nm. Data has been normalised to its maximum.

(B) 293 K absorption spectra of LHCII aggregates. Data has been normalised to its Q_y maximum.

(C) 293 K fluorescence lifetimes of LHCII aggregates. Excitation wavelength was at 468 nm. Emission was measured at 680 nm. Inset value is average lifetime for the trace.

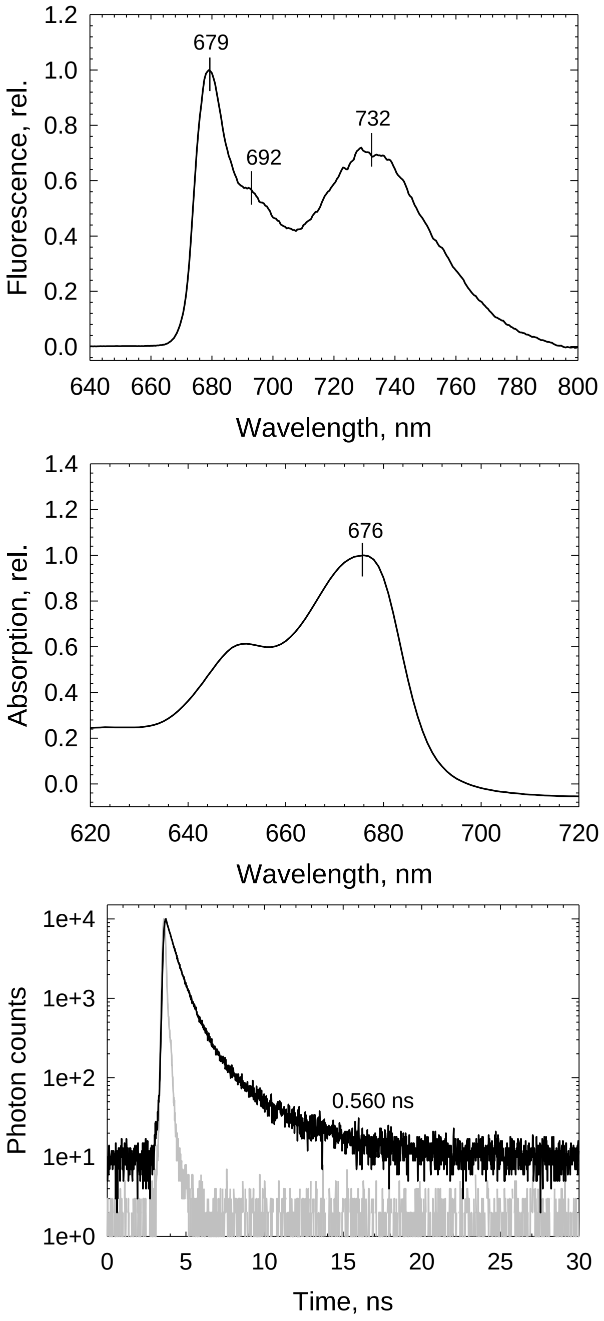

Fig. S7 ***Observation of the F730 band in LHCII incubated overnight in 70% glycerol***

(A) 77 K fluorescence emission spectra of LHCII incubated in a glycerol-rich medium. Excitation wavelength was at 436 nm. Data has been normalised to its maximum.

(B) 293 K absorption spectra of LHCII incubated in a glycerol-rich medium. Data has been normalised to its Q_y maximum.

(C) 293 K fluorescence lifetimes of LHCII incubated in a glycerol-rich medium. Excitation wavelength was at 468 nm. Emission was measured at 680 nm. Inset value is average lifetime for the trace.

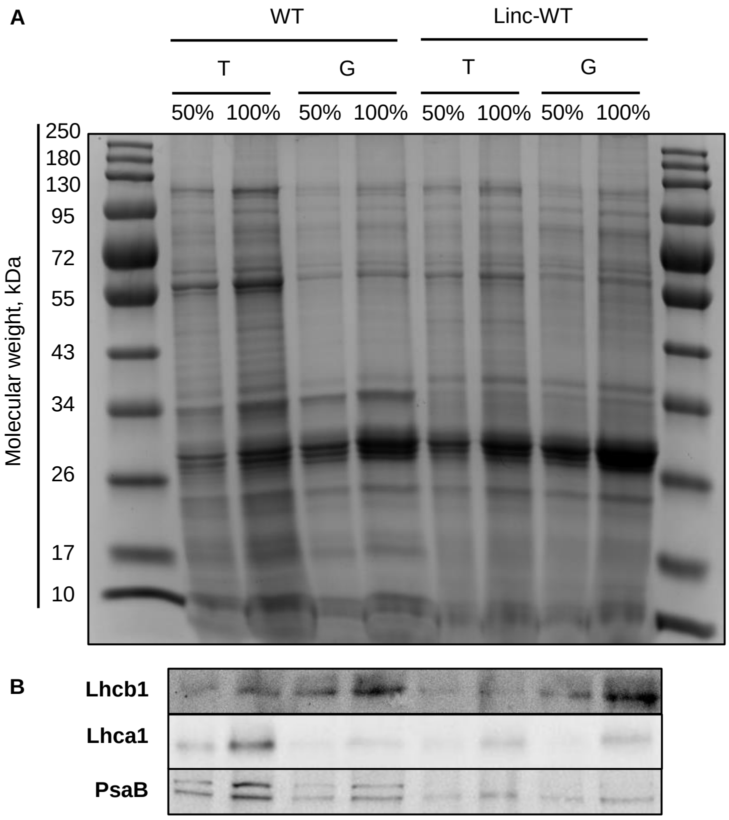

Fig. S8 ***SDS-PAGE and western blot of isolated thylakoids and grana from Arabidopsis WT and Linc-WT***

(A) 12% Tris-glycine SDS-PAGE gel of WT and Linc-WT thylakoid membranes (T) and grana (G) stained with InstantBlue Coomassie protein stain. 100% loaded total chlorophyll was 3 µg.

(B) Western blot for the Lhcb1, Lhca1, and PsaB apoproteins.

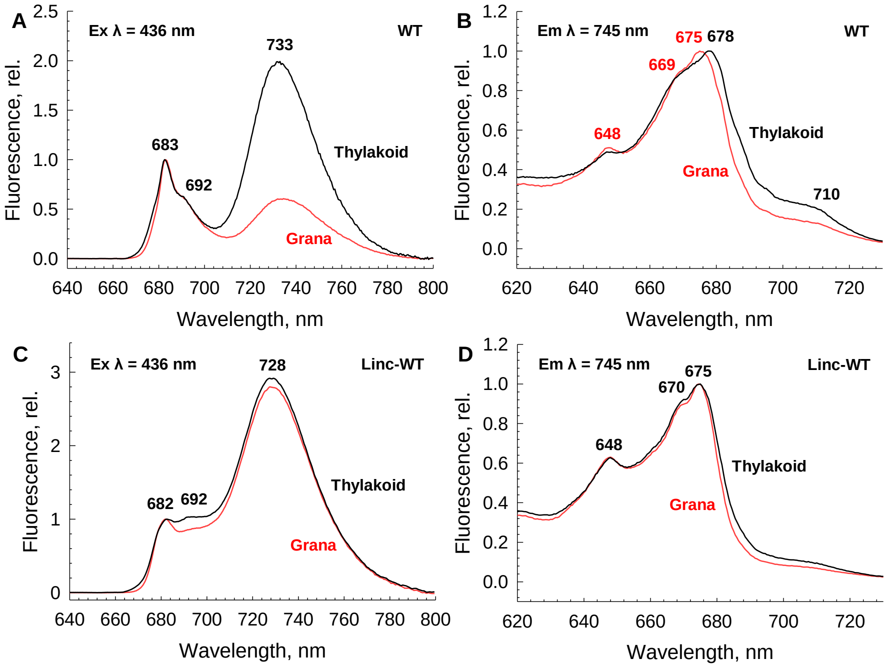

Fig. S9 ***Extended fluorescence spectra of the F730 band in vivo***

(A) 77 K fluorescence emission spectra of WT Arabidopsis thylakoid membranes (black) and WT Arabidopsis grana membranes (red). Excitation wavelength was at 436 nm. Spectra are normalised to their respective maxima ~680-685 nm.

(B) 77 K excitation spectra of WT Arabidopsis thylakoid membranes (black) and WT Arabidopsis grana membranes (red). Emission wavelength was at 745 nm. Spectra are normalised to their respective maxima.

(C) 77 K fluorescence emission spectra of Linc-WT Arabidopsis thylakoid membranes (black) and Linc-WT Arabidopsis grana membranes (red). Excitation wavelength was at 436 nm. Spectra are normalised to their respective maxima ~680-685 nm.

(D) 77 K excitation spectra of Linc-WT Arabidopsis thylakoid membranes (black) and Linc-WT Arabidopsis grana membranes (red). Emission wavelength was at 745 nm. Spectra are normalised to their respective maxima.

		A_1	τ_1	A_2	τ_2	A_3	τ_3	τ_{AVG}
**Chlorophyll-to-lipid molar ratio**	**0.809**	6599	0.2757	9315	0.65851	107.3	3.339	0.519
	**0.794**	7309	0.4243	6989	0.9215	210.9	3.321	0.7059
	**0.767**	3902	0.2657	11785	0.67052	90.2	2.766	0.582
	**0.316**	5026	0.3375	9760	0.81402	95.9	4.022	0.67378
	**0.226**	5205	0.4017	9095	0.9583	79.1	4.744	0.778
	**0.157**	6209	0.5112	7781	1.1278	102.5	4.639	0.8817
	**0.105**	4107	0.5596	9593	1.2645	87	4.083	1.0723
	**0.0897**	2751	0.5785	10368.7	1.5167	168.4	4.002	1.354
	**0.0822**	3521	0.5892	8969.3	1.5322	492.4	3.881	1.3655
	**0.0787**	9.04	62.2	3750	1.1885	8042.8	2.4799	2.1153

Supplementary Table 1 ***Lifetime component analysis for LHCII proteoliposomes with altered chlorophyll-to-lipid ratios***
Amplitude (A) in counts and lifetime (τ) in ns for a range of proteoliposomes shown in Fig. 2.

	Chl *a*:*b*	Neo / 100 Chl	Vio / 100 Chl	Anth / 100 Chl	Lut / 100 Chl	Zea / 100 Chl	β-Car / 100 Chl	Chl *a* / 100 Chl	Chl *b* / 100 chl
B3 (Fig. S2)	1.34 ± 0.02	8.75 ± 0.22	3.02 ± 0.22	N.D.	26.85 ± 1.03	N.D.	2.36 ± 0.47	57.25 ± 0.47	42.75 ± 0.47

Supplementary Table 2 ***Pigment analysis of B3 band via HPLC***
B3 band was taken from Fig. S2 and its pigment composition was analysed via HPLC. Data shown are mean ± SEM (*n* = 4).
